# Exploring a global interpretation mechanism for deep learning networks when predicting sepsis

**DOI:** 10.1038/s41598-023-30091-3

**Published:** 2023-02-21

**Authors:** Ethan A. T. Strickler, Joshua Thomas, Johnson P. Thomas, Bruce Benjamin, Rittika Shamsuddin

**Affiliations:** 1 Physics and Mathematics, East Central University, PO Box 385, Ada, OK 74820 USA; 2 Department of Internal Medicine, Rush University Medical Center, 1700 W Van Buren St, 5th Floor, Chicago, IL 60612 USA; 3 Oklahoma State University, 201 Math and Science Building, Stillwater, OK 74078 USA; 4 School of Biomedical Sciences, Center for Health Sciences, 1111 W. 17th st., Tulsa, OK 74107 USA; 5 Oklahoma State University, 212 Math and Science Building, Stillwater, OK 74078 USA

**Keywords:** Infectious diseases, Computer science

## Abstract

The purpose of this study is to identify additional clinical features for sepsis detection through the use of a novel mechanism for interpreting black-box machine learning models trained and to provide a suitable evaluation for the mechanism. We use the publicly available dataset from the 2019 PhysioNet Challenge. It has around 40,000 Intensive Care Unit (ICU) patients with 40 physiological variables. Using Long Short-Term Memory (LSTM) as the representative black-box machine learning model, we adapted the Multi-set Classifier to globally interpret the black-box model for concepts it learned about sepsis. To identify relevant features, the result is compared against: (i) features used by a computational sepsis expert, (ii) clinical features from clinical collaborators, (iii) academic features from literature, and (iv) significant features from statistical hypothesis testing. Random Forest was found to be the computational sepsis expert because it had high accuracies for solving both the detection and early detection, and a high degree of overlap with clinical and literature features. Using the proposed interpretation mechanism and the dataset, we identified 17 features that the LSTM used for sepsis classification, 11 of which overlaps with the top 20 features from the Random Forest model, 10 with academic features and 5 with clinical features. Clinical opinion suggests, 3 LSTM features have strong correlation with some clinical features that were not identified by the mechanism. We also found that age, chloride ion concentration, pH and oxygen saturation should be investigated further for connection with developing sepsis. Interpretation mechanisms can bolster the incorporation of state-of-the-art machine learning models into clinical decision support systems, and might help clinicians to address the issue of early sepsis detection. The promising results from this study warrants further investigation into creation of new and improvement of existing interpretation mechanisms for black-box models, and into clinical features that are currently not used in clinical assessment of sepsis.

## Introduction

**Figure 1:**
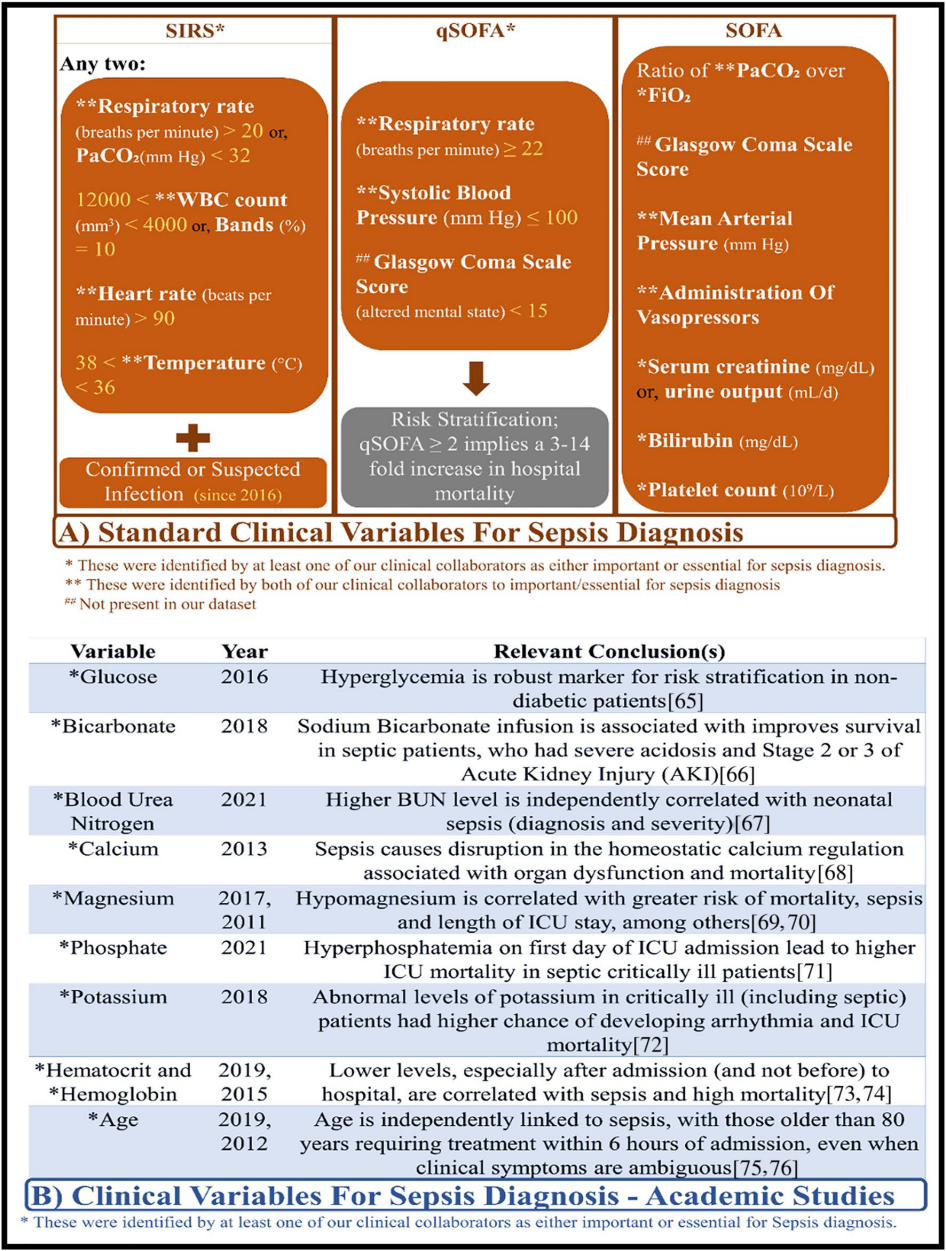
This figure shows the different clinical variables that are known to be affected by sepsis or septic shock. (**A**) Shows the clinical variables used in practice by clinicians to diagnose sepsis. (**B**) Shows the clinical variables that are independently correlated with sepsis and/or those variables specific to certain type of cohort. The “Year” column refers to the year the cited papers were published. The clinical diagnosis scoring ranges are: Systemic Inflammatory Response Syndrome (SIRS) Criteria: (Range, 0–4 Criteria); Quick Sequential [Sepsis-related] Organ Failure Assessment: (qSOFA) (Range, 0–3 Points); Sequential [Sepsis-related] Organ Failure Assessment (SOFA): (Range, 0–24 Points).

Sepsis is a life-threatening organ dysfunction caused by a dysregulated host response to infection^1^. It has a high mortality rate of 6 million per year worldwide, and a healthcare cost of over 16 billion dollars in the USA alone^2–4^. Figure 1A shows the standard medical features and assessments that are currently used to clinically diagnose sepsis. However, an open research question in the sepsis research community is “early sepsis detection”, because by the time these features/assessments provide positive response for sepsis it is often too late for the necessary treatment intervention; and it is known that if a sepsis patient is not treated within six hours of hospital admit time, the mortality rate increases by 9%^3^. This suggests that it is important to find other medical/clinical features that can bolster sepsis detection by clinicians in a timely manner.

Since, machine learning (ML) algorithms are well-known for their ability to discover patterns in data that are otherwise unfathomable to the human eye, PhysioNet hosted a computational challenge in 2019 for sepsis detection^5^. However, despite their ability to optimize, most ML algorithms (especially, the state-of-the-art deep networks) are not good at explaining what it has learnt, let alone elucidate the factors the model used to reach its decision in a human interpretable manner. As a matter of fact, it has been demonstrated that sometimes ML models, with high computational accuracy, can fail to grasp the actual concept (or factors) it was supposed to learn. Thus, human-interpretability of the algorithms need to be addressed for comprehensive use of ML algorithms in sepsis detection to discover additional, relevant, clinical factor.

Currently, with regards to human-interpretability, the ML algorithms can be broadly classified into two categories: (i) ones that are intrinsically interpretable, and (ii) complex, black-box models that are explainable to an extent via the use of post-hoc analysis. Intrinsically interpretable ML models are limited in number, and include linear regression, logistic regression and decision tree; whereas, explainable models consist of black-box models, such as support vector machine (SVM) and deep learning networks (DNNs). Post-hoc analysis of explainable models often relies on an external, model-specific or model-agnostic method that can only provide local explanations of the model. Local explanations only provide information on why any one particular instance was classified a certain way, as opposed to an explanation of what the model has learnt or how much of the model has understood the intended concept (aka. global explanations).

However, the accuracy-interpretability trade-off^6,7^ in ML is still an open research challenge, where model complexity is directly proportional to higher accuracy, but inversely proportional with human-interpretability. Examples of these can also be seen in sepsis detection^8–27^. As a result, even though ML has greatly advanced healthcare data analysis^28–35^, computational healthcare studies (including sepsis detection^8–11,30,36^) often choose statistics, intrinsically interpretable ML models and/or feature selection methods for analysis, instead of state-of-the-art ML models with higher accuracy. Moreover, there is no guarantee that local explanation provided (via post-hoc analysis using LIME^37^/SHAP^38,39^) for one instance in the dataset will be the same for a different instance in the same dataset, even if they share the class membership. As such, features obtained from local explanations will not be a good representation of the additional relevant features needed for timely sepsis detection. Thus, global interpreter for state-of-art-model is essential because it can aid the task of identifying relevant sepsis factors. Moreover, a global interpreter allows black-box models to retain their high accuracy, while becoming more transparent to human beings. Various works have addressed the challenge of creating global interpreters, and some notable examples include^40^, which shows complete equivalency between fuzzy logic and neural networks^41^, which makes use of decision tree structure to train deep networks^42^, which proposes the use of concept vectors over saliency map to ensure the correctness of convolution networks; and Ref.^43^, that uses a deep network to create a decision tree.

In this paper, we propose a post-hoc, model-agnostic interpretable mechanism (IM) to globally understand the sepsis-related concepts learnt during training by a state-of-the-art, black-box ML model. Since, the state-of-the-art models are good at classification tasks, investigating the features they use globally for classification, will be a good indicator for additional features that needs to be investigated for timely sepsis detection. Unlike interpretable solutions presented in Refs.^40–43^, which uses fuzzy rules and decision trees (both are intrinsically interpretable) and visual aids (saliency maps), the proposed IM here leverages the “nearest-neighbor” concept of k-nearest neighbor. Additionally, for human interpretability, it is important to provide explanations of decisions in easy terms that understandable by the general population; yet, some methods such as covariance matrices^2,27^ require a certain expertise for interpretation. Thus, the proposed IM presents the results in a format that is visually and easily understandable by both computational and healthcare personnel via qualitative assessment.

The evaluation of the proposed IM presents an additional challenge, because as of now, there is no standard evaluation technique for assessing “human-interpretability” in the field of ML and computer science. This is further complicated by the fact that literature studies^5,20,21,26,27,30,44–48^, and our own experiments show that it is difficult to obtain high and balanced specificity and sensitivity for ML models on sepsis detection, especially when using the publicly available, physiological dataset^5^ on sepsis; as such, a large body of work on sepsis^16,44–48^, uses text data and Electronic Health Records (EHR).

**Figure 2:**
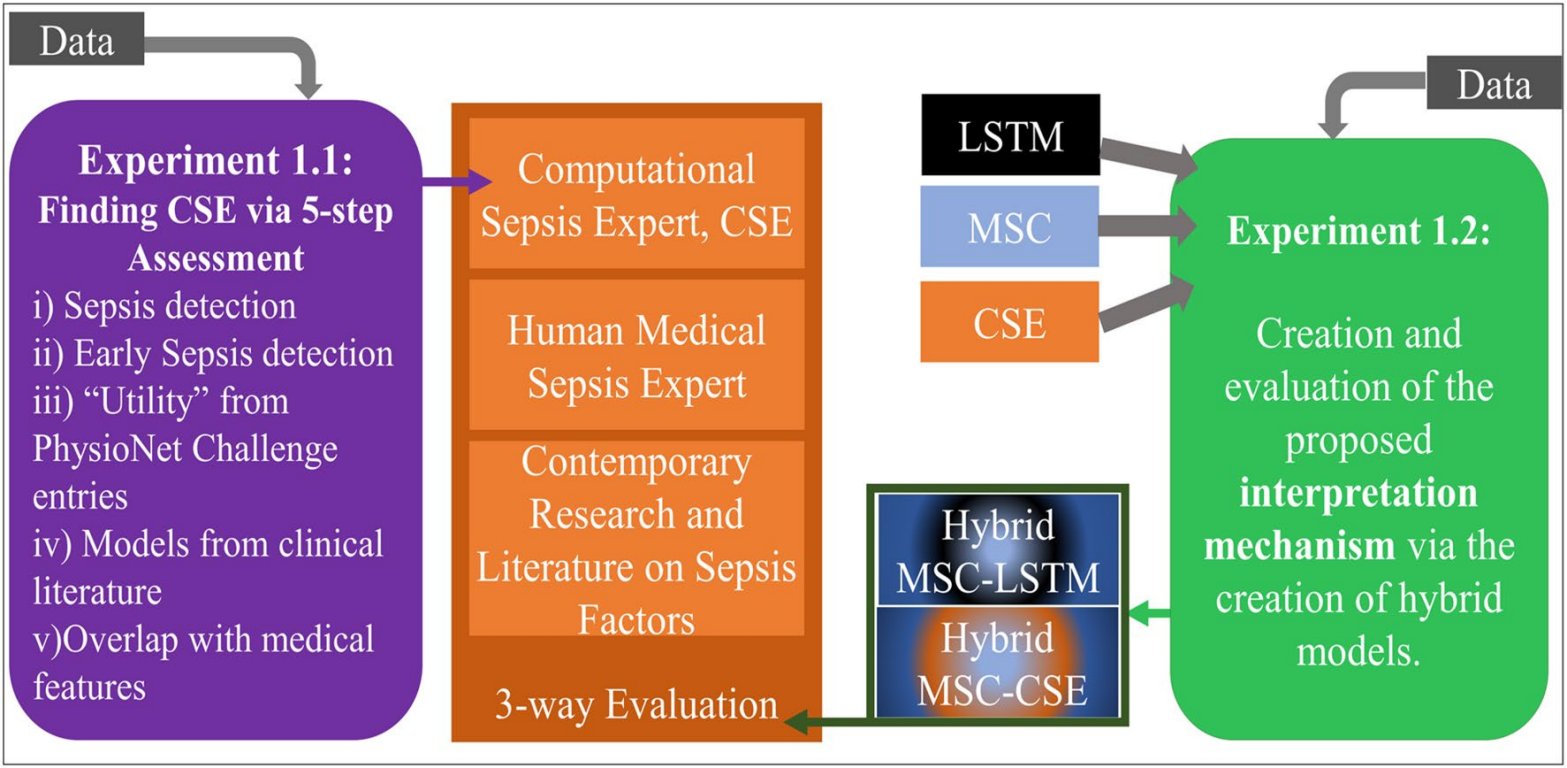
An overview of our approach. Experiment 1.1 is conducted to establish a, computational sepsis expert (CSE) to act as a benchmark in identifying the features a machine needs to “see” to be a good diagnostic model for sepsis. Experiment 1.2 is where we create and assess the proposed interpretation mechanism.

Thus, a second contribution of this paper is the 3-way evaluation scheme proposed to assess the IM, as shown in Fig. 2:

i. Since this is a healthcare research study, it is imperative for the results of the IM to be validated against clinical features and by clinical expert(s). Figure 1A presents the different scoring criteria for sepsis as used in clinical practice, based on our two medical collaborators (co-authors of this paper) and Refs.^49–51^. These factors in Fig. 1A, which we refer to as clinical features, will be used throughout this paper for evaluation.
ii. The clinical features, however, are not the only features that might affect sepsis diagnosis. Medical factors related to sepsis detection are still an ongoing research area, and hence, the IM is validated by academic literature. Figure 1B presents these features that are not used for clinical diagnosis, but still important in treating sepsis patients. We refer to them as literature features.
iii. Last but not least, the IM needs a computational benchmark for comparison of both accuracy and interpretation (local or global). Thus, we experiment with various classes of ML algorithms, and apply a series of assessment to narrow it down to one model, which we call the computational sepsis expert or CSE. As part of the assessment, we also provide a label-shift training paradigm (“CSE determination”).

The remainder of the paper is broadly divided in to two experiments: one for finding and assessing the CSE (Experiment 1.1), and the other for creating and evaluating the IM (Experiment 1.2). We choose the Long Short-Term Memory (LSTM) model as the black-box representative which the IM will interpret. The IM itself is based on the lesser known multi-set classifier, MSC^52^, which can be made intrinsically interpretable semantically and visually using the concept of nearest neighbors. For the CSE, we experiment with convolutional network (Conv1D), support vector machine (SVM), Adaboost, random forest (RF) and MSC. Even though it would have been ideal if MSC was found to be the CSE, our results from the 5-step assessment (Fig. 2) show that RF outperforms most other ML models in our experiments and literature on sepsis detection. RF also satisfies the list of assessment we setup for CSE. The MSC-based IM for the LSTM, while limited by the performance of MSC itself, still identified factors that matched with clinical experts and the CSE. Additionally, the IM supports the expansion of clinical features to include some of the features from literature, while highlighting few other features that are currently not used in clinical assessment or not studied in literature as being directly related to sepsis.

The rest of the paper is organized as follows: we present the dataset description and the preprocessing steps in "Dataset". "Finding CSE and establishing a benchmark (Experiment 1.1)" presents the characteristics we expect CSE to have, and then “CSE determination” list and describe the assessments for finalizing the CSE, with “Implementation details” providing implementation details. “Proposed strategy for explaining black-box ML models globally using MSC” provides a brief description of MSC and the IM creation, with “Evaluation of the IM” providing the evaluation procedure for the IM. "Finding RF as the CSE" illustrates the results from Experiment 1.1 and "Using the proposed IM to explain LSTM" shows the results from Experiment 1.2. Finally, we end with our discussion in "Discussion" and conclusion in "Conclusions".

## Dataset

The publicly available dataset used here is from the 2019 PhysioNet Challenge: Early Detection of Sepsis from Clinical Data^5^. It is an electronic health records dataset sourced from Beth Israel Deaconess Medical Center (hospital system A) and Emory University Hospital (hospital system B). For this dataset, sepsis experts used the Sepsis III guidelines to define sepsis. According to Sepsis III guidelines^1^, sepsis is determined by a two-point change in patient’s Sequential Organ Failure Assessment (SOFA) score accompanied by clinical suspicion of infection. Clinical infection suspicion is determined through the use of IV antibiotic timestamps and blood culture reports. In Ref.^5^, expert description states that “Specifically, if t_suspicion_ – 24 h ≤ t_SOFA_ ≤ t_suspicion_ + 12 h, then t_sepsis_ = min(t_suspicion_, t_SOFA_)”.

**Figure 3:**
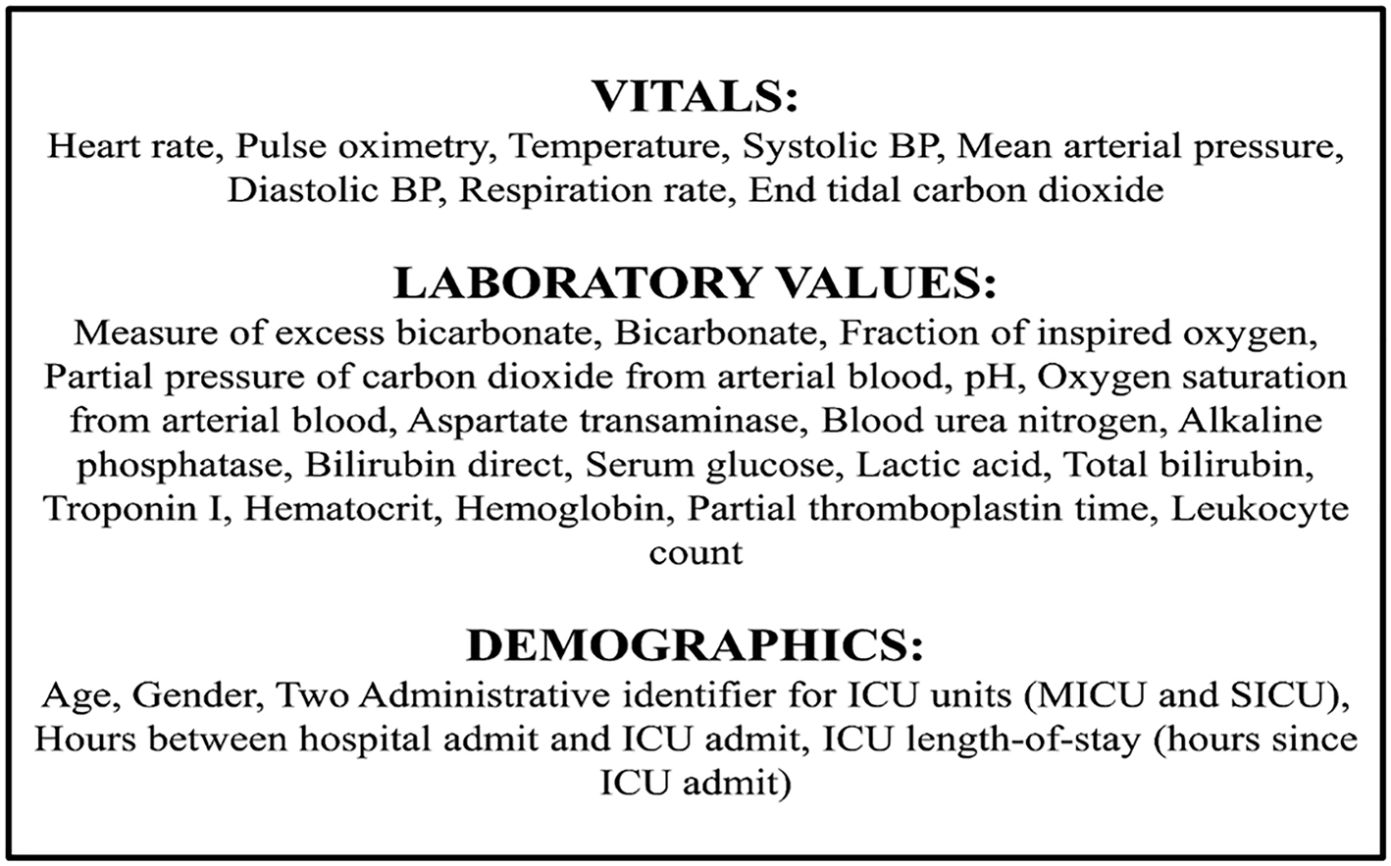
The complete list of sepsis features available for sepsis diagnosis from the 2019 PhysioNet Challenge; more details can be found in Ref.^5^.

The dataset consists of around 40,000 ICU patients with 40 clinical variables for each hour of a patient’s stay at ICU. The 40 clinical variables^5^ can be divided into vital signs (heart rate, blood pressure, etc.), laboratory values (pH, platelet count, hemoglobin, etc.), and demographics (age, gender, etc.). The features are listed in Fig. 3. The data collectors used a combination of patient's SOFA score and time of clinical suspicion (blood culture or IV antibiotics ordered) of infection^1^ to determine whether the patient was septic or not. The dataset has 37,404 non-septic and 2932 septic patients.

### Preprocessing

When possible, for a particular feature, missing values were filled in either by taking the mean of the preceding and subsequent observations, or by using the available value from the most recent past. The remaining missing values were given a value of zero. To distinguish actual data from missing values and to standardize the range of values across the variables, each feature was rescaled to lie between 1 and 6 (inclusive). Since missing values were given the value of 0, the usual [0, 1] range was not an option. Moreover, since there was such a wide variation in the range of the 40 variables in the dataset, we wanted a wider range for re-scaling to obtain better graphs/figures.

## Our approach

As shown in Fig. 2, we organize this paper into two experiments: one for finding CSE ("Finding CSE and establishing a benchmark (Experiment 1.1)"), and another for creating and evaluating the IM ("Creating and Evaluating the IM (Experiment 1.2)").

### Finding CSE and establishing a benchmark (Experiment 1.1)

Any computational diagnostic model, no matter how good, would not have a clinicians’ experience/intuition to make a good call if necessary. For fair evaluation of the proposed IM, instead of just comparing it exactly to clinical features, we should also compare it to what a CSE would use for sepsis classification. Hence, the CSE works as a benchmark against which the results of the proposed IM can be evaluated.

Our search for the CSE is guided by the following characteristic criteria of an ideal CSE. For a machine learning classifier to be considered a sepsis expert:

- The CSE must be good at solving both sepsis detection (detection at clinical diagnosis time) and early sepsis detection (detection prior to clinical diagnosis). “Goodness” can be measured using the following evaluation metrics:
  - Traditional measures: accuracy, specificity, sensitivity, precision
  - Imbalance data measure: F1 score, Mathew’s Coefficient
  - PhysioNet early detection measure: utility^5^. The PhysioNet challenge organizers provided the utility measure as a way to evaluate ML models’ ability to detect sepsis early with only one number.
- When solving the detection task, CSE must demonstrate a good overlap with clinical and literature features (Fig. 1).
  - This can be measured by the intersection of important features by CSE and medical features presented in Fig. 1A. This requires CSE to be interpretable to some extent (or at least explainable through the use of LIME^37^ or SHAP^38,39^).
- We expect the CSE to require the use of more features than those presented in Fig. 1A.
  - The need for these “extra” variables can be justified through the use of Wilcoxon’s Rank Sum test to check for significance and the intersection of these features with literature features in Fig. 1B.

Thus, we adapt the following 5-step assessment (“CSE determination”) to select the CSE from a myriad of literature works and models that we trained.

#### CSE determination

Step 1: Evaluation against ML models using traditional metrics for sepsis detection at clinical time. To identify the model that can achieve best performance on sepsis detection, here we use four representative ML algorithm groups:

- 1D Convolution Network^53^ classifier or Conv1D—this is a state-of-the-art deep network, and requires LIME/SHAP, which can only provide local interpretations. Generally, convolution networks are well-known for finding relevant data representation and often makes use of (i) convolution layers, which select important local features, (ii) batch normalizer, to prompt fast convergence, and (iii) dropout layers, to prevent overfitting. Using rectified linear units (ReLU) helps to address exploding/vanishing gradients, while the softmax activation function for the output layer produces class membership probabilities for the binary classification. Specific implementation details for the Conv1D used in this paper are provided in “Implementation details”.
- AdaBoost^54^ and Support Vector Machine^55^ or SVM—these are traditional ML models and both require LIME/SHAP for local interpretation. AdaBoost models are usually tree-based models.
  AdaBoost is one of the earliest ensemble ML models, which aims to improve classification accuracy by maintain a collection of weal classifiers, each of which is trained to rectify the errors present in the previous learner. This is achieved by updating and re-weighting each instance that was misclassified by the previous learner, such that the subsequent learner prioritizes learning the classification of the previously misclassified instances correctly. It has been shown that this iterative training of weak learners can lower the generalization error and collectively result in a strong classifier. The final classification output is a linear combination of the weighted weak learners:
  $$\mathrm{Y }=\mathrm{ sign}(\sum_{t=1}^{T}{\alpha }_{t}{h}_{t}(x)),$$
  where $\alpha$ is the weight of the learner, h_t_ is the weak learner, and x is the data.
  SVM aims to find a hyperplane or hypersurface that best separates the data in the feature space with support vectors representing the soft margins. The non-linear dual formulation of SVM makes use of LaGrange multipliers and non-linear kernel functions (which can also be represented by semi-definitive Gram matrices) to find an optimal non-linear hypersurface to separate the data in feature space. A detailed derivation of the dual form from the primal optimization problem is outside the scope of this paper. Since SVM optimizes the separating hyper surface, the problem can be formulated as following Langrage function:
  $$L(w,b,\alpha ) = \frac{1}{2}{||w||}^{2 }- \sum_{i=1}^{n}{\alpha }_{i}\left[{y}_{i}h(w,b, {x}_{i})-1\right],$$
  where $\alpha$ are the LaGrange multipliers, and h() is the equation for the hyper surface.

- Random Forest^56^ or RF—this is the most powerful, non-linear model in the class of intrinsically interpretable model that uses the Gini Impurity Index to calculate feature importance. This is a tree-based model, e.g. RF utilizes a collection Decision Trees to arrive at a prediction. Each tree in the “forest” makes a prediction which is then aggregated using techniques of ensemble machine learning models. Each tree learns a non-linear separation of the feature space by optimizing feature threshold values for relevant or significant features. Since each decision tree learns a non-linear boundary, the RFR also learns a non-linear boundary. Assume, that each decision tree is denoted by the function *h*_*i*_(*x,* $\theta$_*k*_), where *h*_*i*_*: x → *[0, 1], and $\theta$_*k*_ is the set of parameters for the model, then an RF can be defined as:
  $$Y =sign((1/N) \sum_{i=1}^{N}{h}_{i}(x, {\theta }_{k})),$$
  where N is the total number of decision trees in the collection.
- Multi-set Classifier^52^ or MSC—this uses concepts of nearest neighbors and unsupervised learning for classification. And in this paper, we show how it can be both conceptually and visually human interpretable. As such, this is the algorithm we propose to base the IM on. A summary of MSC and relevant details are provided in "Creating and evaluating the IM (Experiment 1.2)".

Performance of these models are measured using traditional ML evaluation metrics such as: (i) accuracy, (ii) precision, (iii) specificity, (iv) sensitivity, (v) F1 score, and (vi) Mathew’s coefficient. The model with the best performance on these metrics. e.g. the CSE candidate, is then passed on to the next stage for further assessment.

Step 2: Evaluation of CSE candidate on early sepsis detection (earlier than clinical time) using the proposed label-shift training paradigm. Sepsis detection done × hours before medical determination of sepsis is considered early sepsis detection. Computational detection of sepsis 6 h prior to medical determination is considered optimal, while 12 h prior is considered early^5^.

Given this clinical definition and the computational task for early sepsis detection, we propose the use of a label-shift training paradigm to train the CSE candidate, because none of the models listed in “CSE determination” is as capable as LSTM in capturing temporal patterns. As such we adapt the training paradigm from one of our earlier work^57^ on time series signals, to allow any one of these models to process temporal information. In this training paradigm, instead of trying to optimize model hyperparameters and/or architecture, we re-arrange the data that is being fed to the model.

In other words, the label-shift training paradigm works by leveraging how a ML model learns. Generally, any ML model learns by associating/correlating the information in features (denoted as a feature vector and represented as a row vector) with a particular label (in this case 0 for non-sepsis, or 1 for sepsis). In the sepsis dataset, a patient’s information or test results (e.g. feature vector) is only labeled as 1 after the clinical expert has clinically determined the sepsis diagnosis. However, if we want to detect sepsis early, the ML model needs to associate information from prior test results (e.g. information collected from before clinical diagnosis) with the label 1. As such, if we want to detect sepsis 6 h prior to clinical detection, we first tag all the information collected within that 6-h window as relevant for sepsis determination. This modified information with the tags are then used to train the ML model, inducing them to learn from information that is collected prior to clinical diagnosis. The mathematical description of the tagging process is provided below.

Suppose, each patient is denoted by P_id_, where id is unique to each patient. Each P_id_ contains a set of rows, with the features/attributes separated by different columns. The rows in each P_id_ are temporally dependent such that any two rows, ^id^r_i_, ^id^r_j_ ∈ P_id_, where i < j (and i, j denotes the ith and jth rows in P_id_), can be expressed as ^id^r_t_, and ^id^r_t+c_ respectively, where c is a positive constant, and represents a time interval; for this sepsis dataset, c = 1 h if j = i + 1. The sepsis label associated with ^id^r_t_ is denoted by ^id^l_t_. Thus, the traditional training paradigm dataset is denoted by ^Sepsis^T = {[^id^r_t_, ^id^l_t_] | t ∈ ℤ +}. Under the label-shift paradigm, each ^id^r_t_ gets associated with a label from ‘future’ or subsequent time intervals. The basis for this shift is that we want to the train the ML model to predict at the present time by learning to associate the past data with the future state.

Thus, the training dataset is now denoted by ^Sepsis^T_c_ = {[^id^r_t_, ^id^l_t+c_] | t, c ∈ ℤ +}. Since we are interested in predicting sepsis 12 h, 6 h and 1 h earlier than sepsis onset^5^, we restrict c ∈ [1, 6, 12], resulting in three different training sets. This allows us to measure the performance of the CSE candidate separately for each value of c, using: (i) accuracy, (ii) precision, (iii) specificity, (iv) sensitivity, (v) F1 score, and (vi) Mathew’s coefficient.

Step 3: Evaluation using the utility metric against entries from 2019 PhysioNet Challenge. 2019 PhysioNet Challenge^5^, which is focused on early sepsis detection, provided a new ML evaluation metric, called utility. The utility measures whether the sepsis diagnosis model is able to detect sepsis at an early stage e.g. at most 12 h earlier than the medical expert detection, with 6 h prior medical detection obtaining perfect score. This score takes a minimum value of 0 and a maximum of 1, with higher values indicating better ML model performance. The steps to calculate utility is given in Ref.^5^, and the code is available at https://github.com/physionetchallenges/evaluation-2019.

Since we are using the data that was made available by Ref.^5^, in addition to Step 2, we also compare the utility values of the listed models with the values obtained from top performing entries^5,20–22,26,27,58^ in the challenge^5^.

Step 4: Comparison with sepsis diagnosis models in clinical literature. Due to a need for early sepsis detection, sepsis is a frequently studied medical condition in the medical/clinical field and consists of significant amount of literature work. We identified few of these models tested in clinical setting and compared their performance with the CSE candidate.

The literature models that were chosen are: AISE^30^, the Epic Sepsis Model (ESM)^44^, and the EPIC native sepsis model^45^.

Step 5: Overlap comparison with clinical features. We use the respective explanability/interpretability technique (Gini Index if RF; LIME/SHAP otherwise), to obtain the twenty most important features obtained by CSE to detect sepsis. Their quintiles (maximum, third quartile, median, first quartile, minimum) are then recorded. Additionally, the Wilcoxon’s Rank Sum hypothesis test is calculated using the median for the sepsis and non-sepsis groups to obtain the statistical significance (for all 40 features).

The overlap of these top twenty features with the clinical features and the features from literature (Fig. 1) is then recorded and presented.

#### Implementation details

The convolution model was set up in MATLAB with one 1D convolution model layer (15 filters of size 3 × 3, ReLU activation), followed by a max pooling layer and fully connected layer with softmax activation function. RF was implemented in Python, using 100 estimators, and accounted for the imbalanced dataset by setting built-in hyperparameter “class weight” to “balanced”. AdaBoost was also implemented in Python, using 200 estimators. SMOTE^59^ with “minority” sampling was used to handle class imbalance. For SVM we used the RBF kernel with the regularizer constant set to 0.5, along with in-built mechanism to handle the imbalance in Python. For MSC we used kmeans++ to choose 10 anchors, and set the model to have 1 class profile for sepsis and 1 class profile for non-sepsis, and used the Euclidean distance for measuring similarities.

### Creating and evaluating the IM (Experiment 1.2)

Multi-set Classifier, MSC^52^, is not a commonly used algorithm and relies on nearest neighbor principles for classification. However, its output are class profiles (in addition to class labels), which are low-dimensional data structures^60^. The low dimensionality of the profiles makes the model globally and intrinsically interpretable, and easy to visualizable as bar charts or line graphs. Thus, it is a good candidate for the basis of our IM. Another advantage of MSC is that its choice of anchors is completely data driven and requires no human intervention unlike^47,48^.

#### Proposed strategy for explaining black-box ML models globally using MSC

MSC (full algorithm in Ref.^52^) assumes that each patient/entity is described by a set of feature vectors (instead of one). It begins by using a clustering technique to select a subset of feature vectors called anchors, which act as the base concepts/patterns and are representative of the dataset. Every entity/patient and class profile are then defined as a histogram(s) in terms of these anchors as follows:

##### Overview of MSC

The main idea that motivates the construction of MSC is that even though natural phenomenon, such as sepsis, are complex and non-linear in their details, each phenomenon can be defined by combinations of a set of basic patterns that encompasses or explains the major variations of the phenomenon. Then, understanding a phenomenon or distinguishing one phenomenon from another, boils down to finding this set of representative basic patterns. These patterns are called anchors. In data science and ML, patterns and hence anchors, are represented as vectors. For a medical phenomenon, there are two valid ways of selecting the set of anchors: (i) have one or multiple medical expert manually engineer the anchors (e.g. Figure 1A), or (ii) have a ML model pick the anchors based on the data that is collected on the phenomenon. In this paper, we allow the anchors to be automatically generated anchors from the sepsis via unsupervised ML technique called clustering. There are various methods to perform clustering; and MSC can use any of those techniques to choose the anchors. One advantage of using automated anchor selection is that the user does not need to know specifically which anchor describes sepsis and which anchor describes non-sepsis; this detail is later obtained through analysis of class profiles, which are the output of MSC.

Once the anchors, which represent the phenomena of sepsis and non-sepsis, are chosen via clustering techniques, MSC iterates over each patient individually, recording which anchors are present in that patient, and counting how many times those anchor occur in the patient. MSC organizes this information for each patient as a low dimensional structure, akin to a histogram over the anchors, called a fingerprint. Since the fingerprints are constructed using the frequency count of the anchors, the fingerprint can be regarded as the unique signature of each respective patient for determining sepsis (and non-sepsis) within “them” (or their respective data).

To finally obtain the combinations of the anchors that describe sepsis (and hence, non-sepsis), called the class profiles, fingerprints of similar patients are clustered together and averaged over. Thus, these class profiles represent the anchor combinations that help us distinguish sepsis from non-sepsis. Note, when considering “similar” patients, it makes sense for MSC to keep pool of sepsis and non-sepsis patients separate using the ground truth labels from the dataset. Since the amount of data that is used by MSC is finite the class profile representation of sepsis approximates the actual natural phenomenon.

Since, the pattern vectors and hence the anchors, are composed of health attributes of each patient (Fig. 3), MSC can trace the anchor combinations back to the health attributes, and help us interpret which attributes are responsible for defining sepsis (and non-sepsis) via class profile comparison.

In other words, the basic idea behind MSC is similar to that of Gene Set Enrichment Analysis (GSEA)^61^, where instead of comparing individual genes, a set of genes (related via similar biological pathways) are analyzed together for the phenotype under consideration. Instead of looking as each attribute separately, MSC incorporates their inter-connected behavior through the use of anchors. Thus, the patterns obtained from the set of attributes (aka. the set of genes) are captured by the anchors, which are then used as the building blocks for the class profiles. Each class can then be viewed as a separate biological pathway governed/characterized by different expressions of the genes. And just as GSEA can help to illuminate and interpret complex patterns, MSC has a similar potential to improve human interpretability.

##### Overview of MSC as the IM

Since MSC uses the ground truth label to keep the pool of sepsis and non-sepsis patients separate, the class labels directly impact the nature of the class profiles outputted by MSC. With medical datasets, the ground truth label usually originates from one or more medical experts. However, note that there is no guarantee that two medical experts will agree on all the annotations and/or labels for the data. In other words, each expert “perceives” a different “truth”. Similarly, far into the future, we can also have an AI medical expert diagnosing patients as sepsis or non-sepsis, and this AI expert can have its own perception or understanding of the medical rules when carrying out the diagnosis. Thus, depending on which expert we use to obtain the “ground truth” labels, we will obtain slightly different class profiles, which are biased towards the expert’s perception/view. Similarly, if we obtain the “ground truth” labels from a deep learning model’s predictions, we will get class profiles that conducive to the perception of the deep learning model. Each “source” that is capable of generating ground truth labels for the data in consideration is called an oracle. When the oracle is another ML model, and MSC is used to obtain the perceived truth, it results in a hybrid model. This hybrid model acts an IM.

##### Entity as a collection of concepts

Suppose the set of all feature vectors describing a disease perfectly is called the concept set, and is denoted by Q = {q_1_, …, q_z_, …, q_|Q|_}, where q_z_ ∈ R^d^ and d > 0. Then E_j_, the jth entity in a given dataset, is a member of the power set of Q (excluding the empty set), and is denoted as E_j_ ∈ Power Set(Q)\∅, where ∅ as the empty set.

##### Base concepts or anchors

The anchors are defined as a subset of Q, and denoted by $\widehat{\mathrm{Q}}$ = {*q̂*_i_}, where *q̂*_i_ is the ith anchor. The number of anchors (e.g. |$\widehat{\mathrm{Q}}$|) is determined by the user, following the restriction that 0 <|$\widehat{\mathrm{Q}}$| ≪|Q|. The anchors are chosen using a clustering technique, such as kmeans++, hierarchical clustering, etc. Usually, instead of using the entire dataset, the anchors are chosen from a representation initial sample.

##### Fingerprints

The fingerprint of E_j_, which has a class label k, is a histogram over $\widehat{\mathrm{Q}}$ and written as: ^k^fp_j_ = {^k^p_ji_}, where ^k^p_ji_ is the proportion of *q̂*_i_ present in E_j_.

##### Class profiles

If class k is restricted to having only one profile, then a single profile is the average of all the fingerprints belonging to class k; in our case, k = 1 for sepsis patient and k = 0 otherwise. However, since MSC allows one class to be described by one or more profiles, the set of profiles for the kth class is given by C_k_ = {^r^c_k_}, where r > 0 and indexes the profiles in C_k_. Thus, rth profile of class k is the average of a subset of fingerprints belonging to class k, and is denoted by ^r^c_k_ = {^r^cp_ki_}, where ^r^cp_ki_ is the average of ^k^p_ji_ over a subset of all E_j_ in class k.

##### Building class profiles during training phase

The user determines the number of profiles required to describe each class. The initial sample is used to initialize the class profiles. Then, during the training phase, MSC algorithm executes the following steps to update the class profiles with respect to the feature vectors in each class. During any particular training iteration:

i. A feature vector, maintained in temporal order, is fetched from the dataset. Suppose it belongs to class k (for example k = 0) and patient E_j_.
ii. The algorithm then determines which anchor is most similar to this feature vector. Suppose, it is the best match for *q̂*_m_, then fingerprint, ^k^fp_j_ (or ^0^fp_j_), is updated as follows:
  $$f = ((^{k} p_{{j{\text{m}}}} ) \times n_{j} ) + 1,$$
  $$n_{{\text{j}}} = n_{{\text{j}}} + 1,$$
  $$^{k} p_{{j{\text{m}}}} = f/n_{{\text{j}}} ,$$
  where *n*_j_ is the number of feature vectors seen so far for by the algorithm for entity E_j_.
iii. The updated fingerprint is then compared with the profiles in C_k_ (or C_0_) only to find the closest match. If ^r^c_k_ (or ^r^c_0_) is the best match, then it is updated as follows:
  $$c = (n_{{\text{r}}} \prime \times (^{{\text{r}}} {\text{c}}_{{\text{k}}} )) +^{{\text{k}}} {\text{fp}}_{{\text{j}}} ,$$
  $$n_{{\text{r}}} \prime \, = n_{{\text{r}}} \prime + {1,}$$
  $$^{{\text{r}}} {\text{c}}_{{\text{k}}} = {\text{c}}/n_{{\text{r}}} \prime ,$$
  where *n*_r_′ is number of fingerprints seen so far for by the algorithm for profile ^r^c_k_.
iv. Algorithm fetches a new fingerprint and repeats.
v. Once the class profiles are obtained, testing is achieved by comparing the testing fingerprints to the mature class profiles and recording whether the best matched profile belongs to C_k=0_ or C_k=1_.

Thus, the output of MSC algorithm are labels and class profiles, where each class profile can be a set of sub-profiles. It is important to note that while MSC allows features vectors to change sub-profile membership within a particular class C_k_, it does not allow them to jump between different classes e.g. C_k=0_ and C_k=1_. Thus, traditionally, each class is described by a set of profiles based on information from the ground truth labels, because that’s how the membership of feature vectors is determined during training.

As a result, if we were to randomly change the ground truth for each feature vector during the training phase, the resulting class profiles would be quite different than the ground truth class profiles. Similarly, we can theoretically ask an oracle to provide us with class labels, because either the ground truth is not accessible or the “oracle” sees unexplainable, hidden patterns. These labels may (or may not) be different from the ground truth. Then the class profiles produced by MSC will reflect insights of the pattern seen by the “oracle”. Thus, if a black-box ML model is the “oracle”, e.g. the model’s predictions (instead of ground truth) is used when training MSC, the resulting class profiles will approximate what the black-box ML model sees with regards to the anchors chosen by the MSC algorithm.

#### Evaluation of the IM

A hybrid model is the IM that was created by training MSC with labels from the LSTM or RF as the oracle. For example—in the MSC-LSTM hybrid model, LSTM predictions are used as the ground truth label (e.g. LSTM is the oracle) and MSC is used to obtain the class profiles. Similarly, in the MSC-CSE hybrid model, CSE predictions are used as the ground truth label (e.g. RF is the oracle) and MSC is used to obtain the class profiles. Thus, explanation from the MSC-LSTM hybrid model can be taken as approximation for what the LSTM sees, while the MSC-CSE hybrid model is for comparison purposes.

Even though the hybrid models are not the actual predictive model for sepsis, we still need to take their accuracy and fidelity into account because these values provide insight into how well the interpretable hybrid models can explain the black-box models. The fidelity^43^ of the MSC-hybrid models is calculated by recording consensus in classification between the LSTM/RF model(s) and their respective hybrids. Fidelity is calculated in the same way as accuracy, but instead of using the ground truth, we use the labels from the oracle.

In addition, we compare the sepsis profiles obtained from the hybrid models to identify what features the LSTM and RF models are looking at for sepsis classification. We present all our results as comparisons between:

- MSC-LSTM versus MSC-CSE model (profile comparison to obtain what the LSTM learnt)
- MSC-CSE versus CSE (feature overlap, including clinical and literature features)
- MSC-LSTM versus CSE (feature overlap, including clinical and literature features).

##### LSTM overview

LSTM^62^ are deep neural network structures that uses feedback loops and gates to retain long-term temporal dependencies in data. This makes LSTM models suitable for learning and processing sequential data such as the sepsis data. The LSTM layer is composed of the LSTM module, where each time point is processed by a single LSTM module, and the output of this module feeds into the next time point. Each module consists of three gates, the forget gate, input gate, and the output gate. The forget gate decides whether to remember and let past information pass; the input gate combines information from incoming data with past information; and the output gate regulates the final output of the module. The gates are guarded by the sigmoid function; a value of 1 allows information to flow through, whereas a values of zero blocks information passage or facilitates past information loss.

#### Implementation details

The LSTM model was set up in MATLAB with one LSTM layer of 100 hidden units, followed by a fully connected layer with softmax activation function. For this experiment, we use the LSTM as the black-box model that is globally explained by creating a hybrid using its predicted labels to train MSC (as described in “Proposed Strategy for Explaining Black-box ML Models Globally Using MSC” and “Evaluation of the IM”). The MSC is also trained using the ground labels and labels from CSE (to get the benchmark hybrid model). All three models are evaluated using (i) accuracy, (ii) specificity, (iii) sensitivity, (iv) precision, (v) F1 score, (vi) Mathew’s coefficient and (vii) fidelity metric.

Additionally, choosing the anchors are crucial to the creation of class profiles. MSC chooses the anchors via kmeans++ clustering. However, the results of clustering vary based on the number of clusters set by the user. Thus, to address this point, when choosing the anchors, we run the clustering nine times, each time increasing the number of clusters by one. Currently, MSC starts with two clusters and goes up to ten. For each clustering iteration, the silhouette coefficient—a metric that measures the quality of the resultant clusters—is calculated before moving on to the next iteration. The coefficient is 1 for good quality cluster, 0 for indifferent and − 1 for bad. The cluster number that attains the maximum silhouette coefficient is retained, and the respective cluster centers used as anchors.

## Results

In this section, we present our findings and Table 1 summarizes the results of our experiments.

**Table 1:** This table summarizes our results and shows the breakdown of the top 20 (out of 40) features found by the CSE to be relevant for sepsis detection. These 20 features are then used to compare the features found by the IM with (i) clinical features, (ii) features from literature, and (iii) features from human medical experts (marked with single and double asterisks). Note, a single asterisk means that the feature is chosen by at least one of our medical expert; while double asterisks mean that the feature is chosen by both medical experts. The table also shows relevant features that the CSE and LSTM used for the sepsis classification task, but currently not regarded as important in the sepsis medical community. The overlap between the CSE and IM is also displayed (underlined).

	Clinical features	Medical literature features	Other possible relevant features	Top 20 feature overlap
RF, CSE	Platelets*, Temperature**, White blood cell/Leukocyte count**, Fraction of inspired oxygen (FiO_2_)*, Heart rate**, Systolic blood pressure**, Respiration rate**, Mean arterial pressure**, Creatinine* Total: 9	Age*, Hemoglobin*, Hematocrit*, Glucose*, Blood urea nitrogen*, Bicarbonate*, Potassium* Total: 7	ICU length-of-stay, hours between hospital and ICU admit, Diastolic blood pressure**, Chloride Total: 3	(1) Clinical and literature features overlap with RF = (9 + 7) × (100/20) = 80% (2) Overlap with medical expert opinion = (17/20) × 100 = 85%
LSTM (IM hybrid)	Partial carbon dioxide pressure arterial blood**, Fraction of inspired oxygen (FiO_2_)*, Platelets*, Creatinine* Total: 4	Glucose*, Blood urea nitrogen*, Bicarbonate*, Calcium*, Magnesium*, Phosphate*, Age*, Hemoglobin*, Hematocrit*, Potassium* Total: 10	pH, Diastolic blood pressure**, Oxygen saturation Total: 3	(1) Clinical and literature features overlap with IM = (4 + 10) × (100/20) = 70% (2) RF, CSE overlap with IM (underlined) = 11 × (100/20) = 55% (3) Overlap with medical expert opinion (marked with asterisk) = (15/20) × 100 = 75%
Not in dataset	Glasgow Coma Scale		n/a	

Using RF as the CSE, we identify the top twenty features that computationally helps the CSE to detect sepsis. These twenty features are then used to compare the effectiveness of the proposed IM on LSTM through comparison with each other and with clinical and literature features (Fig. 1). The empirical distribution information (maximum, third quartile, media, second quartile, and the minimum) of these twenty features are presented in Table 1.

The explanation for finding RF as the CSE using the 5-step assessment (described in "Finding CSE and establishing a benchmark (Experiment 1.1)") is detailed in "Finding RF as the CSE.". Additionally, "Using the proposed IM to explain LSTM" provides more details about how the IM is used to obtain important features used by LSTM for sepsis classification, and its efficacy and limitations.

For all ML models, the dataset underwent a 70–30% split, with 30% reserved for testing. In-built methods from the sklearn Python library was used to cross validate the model using the training set using tenfold splits and 3 repetition cycles. All results for ML evaluation metrics, presented in this section, are those obtained from the test set.

### Finding RF as the CSE

#### Finding the CSE candidate

The observation here is that despite accounting for imbalances in the dataset, none of the models have a balanced performance across all the evaluation metrics (Table 2). Regardless, RF obtains the highest accuracy, precision, specificity, F1 score and Mathew’s coefficient but have very low sensitivity (Table 2). Additionally, since RF can use Gini Index for global interpretation and does not require a local explainer, we choose RF as the CSE and implement the label shift training paradigm on RF.

**Table 2:** Different evaluation metric values for the different machine learning models from Step 1 of Experiment 1.1. The highest value for each column (aka. evaluation metric) is boldfaced.

	Accuracy (%)	Precision (%)	Specificity (%)	Recall/sensitivity (%)	F1 score	Mathew’s coefficient
Conv1D	81.5	74.4	63.8	63.8	0.66	0.42
RF	**99.0**	**92.5**	**99.9**	56.3	**0.70**	**0.92**
SVM	85.0	9.7	85.0	**74.4**	0.17	0.23
AdaBoost	88.0	10.2	88.3	60.8	0.17	0.21
MSC	67.5	50.5	66.3	69.8	0.58	0.34

Highest values obtained are in bold.

#### Applying the label-shift paradigm to RF

Since RF does relatively better than other models presented in Table 2, we apply the label shift paradigm to RF. The result, in Table 3, shows that RF does pretty well for early detection of sepsis (prediction ranging from 1 to 6 to 12 h prior clinical determination) with comparable performances, and attains the best performance at 12-h prior sepsis detection. Since SVM has highest recall in Table 2, we also tested SVM for early sepsis detection but it did not perform as well RF (and hence, the results for SVM are not presented in Table 3 for readability).

**Table 3:** RF accuracy measures for early sepsis detection. The results from Table 2 are included in this table as the “no shift” (e.g. zero shift) to put the sepsis detection at clinical determination time versus early sepsis detection in context. Utility is the additional evaluation metric^5^ that measures how well the model is doing at early sepsis detection.

	Accuracy (%)	Precision (%)	Specificity (%)	Recall/sensitivity (%)	F1 score	Mathew’s coefficient	Utility
No shift (Table 1)							
RF	**99.0**	92.5	**99.9**	56.3	0.70	0.92	0.83
1-Hour shift							
RF	**99.0**	93.6	99.8	60.4	0.73	0.93	0.82
6-Hour shift							
RF	**99.0**	95.8	99.8	71.4	0.82	0.95	0.89
12-Hour shift							
RF	**99.0**	**97.0**	99.8	**78.8**	**0.87**	**0.96**	**0.88**

Highest values obtained are in bold.

#### Comparing RF utility with entry models from PhysioNet Challenge

In the 2019 PhysioNet Challenge^5^, which had a total of 104 teams from academia and industry (out of which only 88 qualified), the top five submissions had average utility scores of 0.4260^26^, 0.4105^5^, 0.4085^20^, 0.4025^21^, and 0.4025^27^ on the dataset from the two hospitals used for this paper. Table 3 shows that the RF had better utility compared to the entries. However, a word of caution—a team^22^, using a XGBoost model with a Bayesian optimizer and an ensemble learning framework obtained a utility of 0.522 on the two public datasets, but the utility dropped to 0.364 when the model was tested on a hidden dataset from a third hospital.

#### Comparison with sepsis diagnosis models from existing medical literatures

Based on literature search, one good candidate for the CSE could have been AISE^30^, which achieved an AUROC value between 0.83 and 0.85, an accuracy of 72% (maximum) and a sensitivity of 85% and specificity of 67%. However, AISE’s accuracy drops to 60% as it moves from 0 to 12-h window; whereas, the RF trained under our proposed training paradigm (12-h shift) has an accuracy of 99%, precision of 97%, specificity of 99.8% and a recall/sensitivity of 78.8%. In addition, RF reaches a maximum utility 0f 0.89 under 6-h shift, which drops only slightly to 0.88 as we move from 6-h to 12-h shift; even the No-shift RF achieves a utility of 0.83. This shows that RF is better at early sepsis detection.

Another candidate could have been the Epic Sepsis Model (ESM)^44^, a proprietary sepsis prediction model, but^44^ concludes that “This external validation cohort study suggests that the ESM has poor discrimination and calibration in predicting the onset of sepsis. The widespread adoption of the ESM despite its poor performance raises fundamental concerns about sepsis management on a national level.” This conclusion excludes the Epic Sepsis Model as a CSE. But to be fair, our paper and^44^ are using different datasets, and the RF model in this paper was not externally validated.

A third candidate for CSE could have been the EPIC native sepsis model^45^. However, the reported evaluation metric values for the model used in Ref.^45^ lies in the 33–78% range.

#### Comparison with clinical features

Table 4 presents statistical description for the top twenty RF features (relative importance > 0.02) in order of importance, with ICU (Intensive Care Unit) length-of-stay having the highest relative importance of 0.146.

**Table 4:** Statistical description for the top 20 features from RF (zero-shift) model. Q1 and Q3 stands for the first and third quartiles. The minimum, first quartile, median, third quartile and the maximum of a variable are often used as an empirical distribution approximation. Note that a minimum of 0 refers to missing values.

	ICU length-of-stay	Platelets	Tempera-ture	Age	Leuko-cyte count	Fraction of inspired oxygen	Heart rate	Hours between hospital and ICU admits	Systolic blood pressure	Hemo-globin
Sepsis										
Maximum	6	**4.026**	**5.6107**	6	**3.659**	**1.5**	6	6	5.9821	**4.809**
Q3	**2.2687**	1.6032	4.8274	4.7791	1.1648	1.25	2.7801	5.9679	3.2008	2.5101
Median	**1.6269**	1.3993	**4.5929**	4.1395	1.1194	**1.063**	2.4423	5.9678	2.8533	2.2752
Q1	1.2388	**1.196**	2.75	3.3256	**1.076**	0	2.1538	5.9085	2.5444	**2.04**
Minimum	1	0	0	1.0233	0	0	0	**1.762**	0	0
Non-sepsis										
Maximum	6	**6**	**6**	6	**6**	**6**	6	6	6	**6**
Q3	**1.4627**	1.5231	4.7359	4.722	1.133	1.062	2.7277	5.9919	3.1622	2.5268
Median	**1.2836**	1.3274	**2.925**	4.0233	1.0864	**0**	2.3846	5.9678	2.8436	2.2094
Q1	1.1343	**0**	2.65	3.2674	**0**	0	2.1257	5.9336	2.5714	**0**
Minimum	1	0	0	1	0	0	0	**0**	0	0

	Hematocrit	Respira-tion rate	Mean arterial pressure	Creati-nine	Glucose	Blood urea nitrogen	Bicarbo-nate	Chloride	Diastolic blood pressure	Potas-sium
Sepsis										
Maximum	5.5745	6	5.9643	**3.433**	6	**4.774**	5.9303	6	5.8571	**4.517**
Q3	3.0468	2.4706	2.2222	1.1505	1.7311	1.618	3.2727	4.4034	1.8813	1.8621
Median	2.743	2.1111	2.0185	1.086	1.593	1.3396	**2.727**	**4.067**	1.6964	1.6038
Q1	**2.4124**	1.8586	1.8426	**1.052**	1.4727	**1.187**	0	0	1.5216	**1.509**
Minimum	0	0	0	0	0	0	0	0	0	0
Non-sepsis										
Maximum	6	6	6	**6**	6	**6**	6	6	6	**6**
Q3	3.0393	2.2626	2.2143	1.1075	1.6876	1.3774	3.0909	4.2353	1.875	1.8621
Median	2.6465	2.0101	2.0179	1.0645	1.5368	1.2075	**0**	**0**	1.6786	1.6038
Q1	**0**	1.8088	1.8393	**0**	1.3783	**0**	0	0	1.4137	**0**
Minimum	0	0	0	0	0	0	0	0	0	0

Significant values are in bold.

Comparing Fig. 1A and Table 4, we find that all clinical features in Fig. 1A, with the exception of bilirubin and Glasgow Coma Score (not present in the dataset), appear in the top twenty features used by RF to classify sepsis, thus, confirming a good overlap e.g. 12 out of the 14 clinical features were in the top twenty RF features for sepsis detection. While RF also uses bilirubin for classification, it does not appear in the top twenty features. RF also shows an excellent overlap with features in Fig. 1B (summarized in Table 1).

Moreover, the Wilcoxon’s Rank Sum hypothesis test for these top twenty features showed significant median difference at an error rate of 5%. The associated p-values were 0.000, except for “mean arterial pressure” (p-value = 0.0479). Among the forty variables, “phosphate”, “partial thromboplastin time”, “gender”, and the two different ICU units were insignificant with p-values 0.2796, 0.6579, 1.000, 1.000 and 1.000 respectively. Thus, this further ensures that the features used by RF, even if not used in clinical assessments (SIRS, SOFA, qSOFA), are indeed important for features for computationally identifying sepsis.

After examining the results obtained from performing the 5-step assessment described in "Finding CSE and establishing a benchmark (Experiment 1.1)", we move forward with RF as the CSE.

### Using the proposed IM to explain LSTM

Table 5 shows the computational evaluation metric and fidelity of the hybrid models and the individual models themselves. We find that even though MSC has good utility (compared to the highest utility score of 0.4260 reported in Ref.^5^), its accuracy is not as high as RF, and that limits the performance of the hybrid models as well. While ideally, we want the accuracy and fidelity values as close to one another as possible for model explanation, that is hard to come by in practice. Thus, going forward, we need to keep in mind that the hybrid models will explain at least 50% (fidelity) and at most 67% (accuracy) of the concepts learnt by RF and LSTM.

**Table 5:** Predictive performance of the hybrid models on the same test set.

	Accuracy (%)	Fidelity (%)	Precision (%)	Specificity (%)	Recall (%)	F1-score	Mathew's coefficient	Utility
MSC	67.5	n/a	50.5	66.3	69.8	0.58	0.34	0.51
RF	99.0	n/a	92.5	99.9	56.3	0.70	0.92	0.83
LSTM	91.1	n/a	81.4	74.1	74.1	0.77	0.42	n/a
MSC-RF	67.1	53.6	49.9	66.3	68.9	0.58	0.33	0.51
MSC-LSTM	67.9	53.0	46.9	65.4	73.9	0.57	0.36	0.51

#### Hybrid-LSTM versus hybrid-CSE

**Figure 4:**
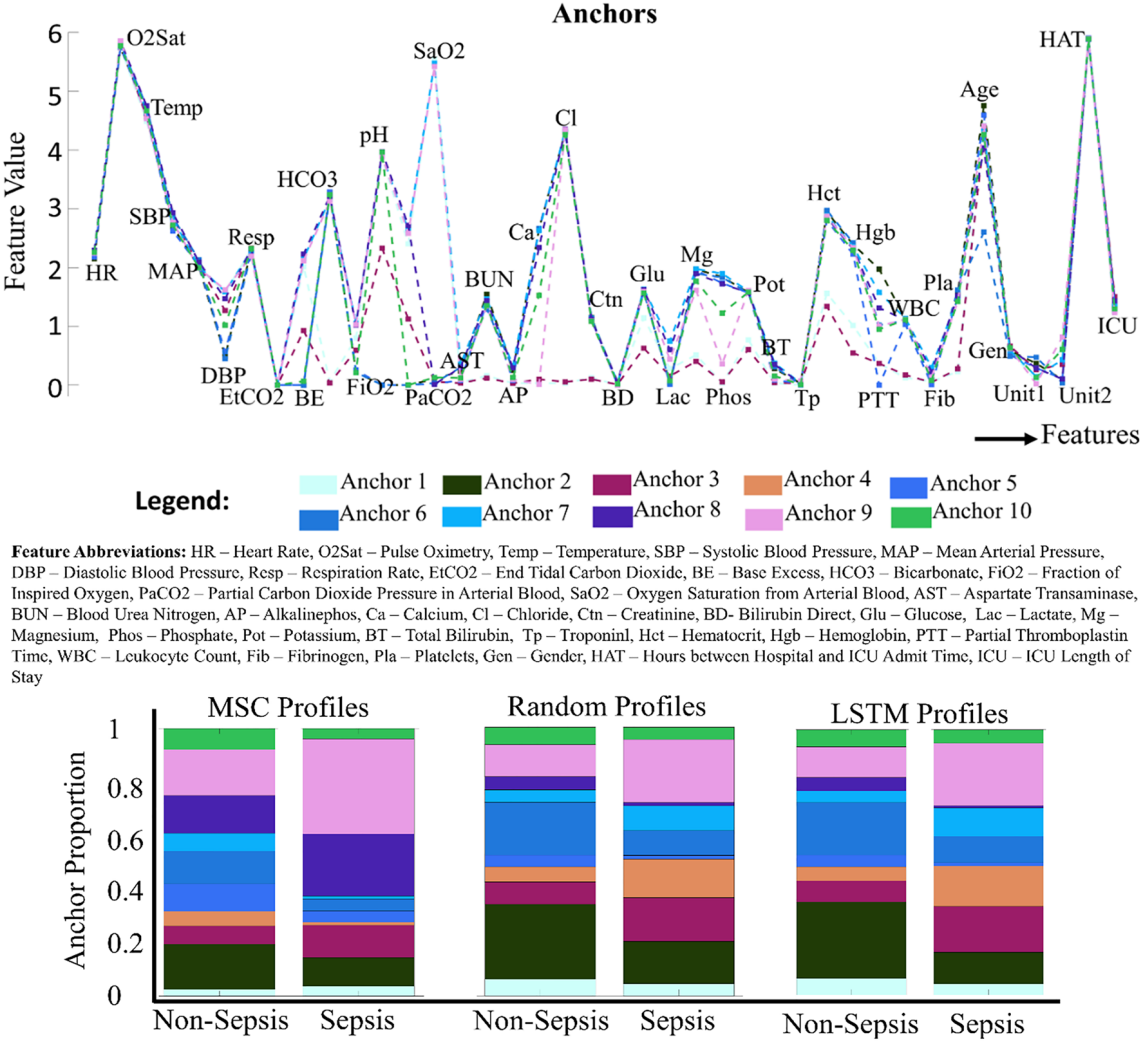
The MSC model and the hybrid models all use the same anchors illustrated and overlaid here to allow valid comparison. The sepsis (and non-sepsis) class profiles created from these 10 anchors by MSC, MSC-RF, MSC-LSTM models, respectively are also displayed. While the profiles for each class for both hybrid models look similar, they differ significantly from the sepsis profiles obtained from MSC.

Figure 4 shows that the profiles from MSC-RF and MSC-LSTM not only look similar but are similar to the first decimal number (even though they differ from the MSC profiles in major ways). This is expected because both RF and LSTM individually as an accuracy over 90% (Table 5).

Concepts of sepsis as seen by RF and LSTM through the eyes of the hybrid models: For the two hybrids, from Fig. 4, we find that:

- Anchors 3 and 9 occur in higher proportions in sepsis group, while Anchor 2 occurs in higher proportions in non-sepsis, indicating that main features for differentiating sepsis (as seen by the hybrids) are:
  - pH, partial carbon dioxide pressure from arterial blood, diastolic pressure, fraction of inspired oxygen, glucose, platelets, bicarbonate, blood urea nitrogen, calcium, magnesium, phosphate, potassium, hematocrit, hemoglobin, and creatinine. Most of these features matches those in Fig. 1, and were found to be significant by Wilcoxon’s Rank Sum test (Experiment 1.1). However, due to lower portions of Anchor 8 and when combined with Anchors from 1, 2, 3, 9, and 10, it seems to suggest that the hybrids see pH, partial carbon dioxide pressure and oxygen saturation as necessary but not very common features.
- Anchor 6 is high in proportion for the non-sepsis class. The main difference between Anchor 6 and all the other anchors is:
  - The lower value for the “age” variable. This is supported by Fig. 1B and Table 4, which show that the non-sepsis group tend to have a lower age. Also, the Rank Sum test (done in Experiment 1.1) found “age” to be a significant variable.
- Anchors 4, 7 and 9 are very similar pattern-wise, but Anchor 9 displays a wider range of variable values. Thus, even though these anchors do not inform about classification explanation, higher proportions of Anchor 9 in the hybrids (compared to MSC) might point to the ability of LSTM and RF to represent finer details than the MSC models.
- Anchors 1, 5 and 10 are not informative in terms of sepsis differentiation, and seems to capture the common patterns present in both classes.

#### (Hybrid-RF, Hybrid-LSTM) versus RF

According to the IM, for sepsis classification, the LSTM and the RF models are using pH, partial carbon dioxide pressure from arterial blood, age, diastolic pressure, fraction of inspired oxygen, glucose, platelets, bicarbonate, blood urea nitrogen, calcium, magnesium, phosphate, potassium, hematocrit, hemoglobin, and creatinine. 11 of these 17 features coincide with the top twenty features from RF (Table 1). In addition, similar to the CSE, the IM tells us that the LSTM model consider “age” to be important. The LSTM model also places some importance on pH, partial carbon dioxide pressure and oxygen saturation (not in the top twenty RF features). We also find that unlike the RF CSE, the IM does not identify ICU length-of-time, temperature, systolic blood pressure, interval between hospital and ICU admits, heart rate, leukocyte count etc. (Table 1) as important features.

## Discussion

In this paper, we propose the development of a data-driven, semi-automated IM for qualitatively evaluating concepts learnt by black-box ML models. This IM is designed to mitigate the prevalent issue of accuracy-interpretability trade-off^6,7^ in machine learning, while addressing the needs of transparency in healthcare, and is tested on a LSTM model to aid timely sepsis diagnosis.

The strength of this work is three-folds. One, it shows that it is feasible to create an IM using the nearest neighbor concept, in addition to the use of decision trees and fuzzy rules; though better accuracy for MSC is desirable. Two, it presents an evaluation method for the IM using clinical and literature through the establishment of the CSE, specifically for sepsis detection. And three, we report features that are not currently considered by the sepsis medical/research community, but might aid in timely sepsis detection.

By reinforcing the use of anchors (which are selected based on the nearest neighbor and clustering concepts), MSC ensures that the IM’s output can be interpreted in terms of clinical features (as opposed to the data representation from black-box models that remains uninterpretable to a human, or covariance matrices that requires statistical knowledge). Moreover, the choice of anchors is data driven and does not require human/expert intervention; however, should the need arise, MSC can also incorporate anchors selected by medical experts. By compressing the complex data into low-dimensional structures, MSC allows the proposed IM to produce outputs in form of bar-chars or line graphs, which further serves to make the results interpretable to a wider population.

The establishment of the CSE (through the use of the proposed label-shift paradigm and literature comparisons), which had excellent overlap with clinical features (Table 1), showed that machine learning models are not just computational optimizers; but rather, ML models have the ability to pick up trends in clinical features. The CSE also showed that literature features, even when independently linked to sepsis, can be useful features for sepsis (early) diagnosis. This confirms our belief that even while lacking a clinician’s experience, if given enough relevant features (including features not used in standard clinical diagnosis) and data, ML models can aid clinicians in real-life with making decisions. With black-box ML models reigning the frontiers of data analysis, the development of an IM that can work independently of model type, can revolutionize healthcare systems.

In this paper, out of the total 40 features present in the dataset, we studied the top 20 features from RF CSE, and the 17 features from the proposed IM (Table 1). Among these 20 and 17 features, there was an overlap of 11 features. Both the RF CSE and the IM agree that features found in medical literature (such as age, hemoglobin, hematocrit, glucose, blood urea nitrogen, bicarbonate, potassium, etc.) but not used in SIRS, SOFA or qSOFA are important for sepsis detection. Other features that were unique to the RF CSE are: ICU length-of-stay, hours between hospital and ICU admit, and presence of chloride ions. Features unique to the IM are: pH and oxygen saturation. Based on medical expert opinion, treating clinicians do not find it ethical or reasonable enough to use “age”, “length of ICU stays” and/or “hospital admit time” to “discriminate” between patients and their treatment plan; even though older patients have been shown to be more susceptible to sepsis^63,64^. Thus, features that can be relevant in clinical settings and thus warrants further investigation are: chloride ions, pH and oxygen saturation. In addition, further investigation into “age” is warranted because not only medical literatures^63,64^, but also both the RF CSE and the IM found that even though patient of any age can develop sepsis, older people have more to lose.

The reason for a lack of a 100% overlap between the RF CSE and the IM might be attributed to the difference in accuracy seen between them in Tables 2 and 5. Another possible explanation, based on the opinion of our clinical collaborator, maybe that strong correlations exist between: (i) pH and systolic blood pressure, (ii) partial carbon dioxide pressure and respiratory rate, and (iii) oxygen saturation, fraction of inspired oxygen, and respiratory rate. Since the LSTM (or the IM) picks up on fraction of inspired oxygen, pH, partial carbon dioxide pressure, and oxygen saturation, maybe it finds that respiration rate and systolic blood pressure no longer results in further gain in new information.

However, there are also limitations to our current study. One limitation of the proposed methodology is that MSC has a much lower accuracy than either RF and LSTM, and so the fidelity is limited by MSC’s reduced discriminative abilities. Two, as implemented in this paper, even though we were able provide interpretation of the LSTM model qualitatively and used indirect quantitative measures for evaluation, a direct quantitative measure is still lacking. Three, none of the models used here had been externally validated (a key requirement to use ML models in clinical settings). However, this study still warrants further investigation into the development of IMs and using the concept of nearest neighbors for aiding black box ML model to attain transparency. As such, we hope to address these three limitations in our future work.

## Conclusions

In this paper, we investigated the interpretability of complex ML models through creation of interpretable hybrids using MSC. Using the proposed label-shift paradigm, five ML models, literature comparison and seven evaluation metrics (including one for measuring early sepsis detection), we found Random Forest to be a good computational expert for sepsis diagnosis. Next, we used MSC (the base of our proposed IM) to create hybrid models for Random Forest and LSTM to gain insight into what the two models have learnt regarding sepsis. Both hybrid models showed significant feature overlap with the CSE and the clinical features. The results of Wilcoxon’s Rank Sum test also supported the identified features by hybrids as features that plays a crucial role in recognizing sepsis. The results presented here show great promise for continued use and further exploration of MSC for unraveling black-box ML models for healthcare studies.

## Data Availability

The datasets generated and/or analysed during the current study are available in the PhysioNet Library repository (PhysioBank), https://archive.physionet.org/pnw/challenge-2019-request-access.
